# Solvent Retention Capacities of Oat Flour

**DOI:** 10.3390/ijms18030590

**Published:** 2017-03-13

**Authors:** Qianwen Niu, Yu Pu, Xiaoping Li, Zhen Ma, Xinzhong Hu

**Affiliations:** College of Food Engineering and Nutrition Science, Shaanxi Normal University, Xi’an 710119, China; niuqianwen@hotmail.com (Q.N.); buchenxiao@126.com (Y.P.); xiaopingli@snnu.edu.cn (X.L.); freyazhen@hotmail.com (Z.M.)

**Keywords:** β-glucan, solvent retention capacities (SRC), CaCl_2_, damaged starch, oat flour

## Abstract

This study measured the solvent retention capacities (SRCs) of flours from eight oat varieties and one wheat variety against different solvents to explore the swelling volume of oat flour with different solvents, and thus provide a theoretical basis for quick β-glucan analysis. The SRC profile consists of water SRC (WSRC), 50% sucrose SRC (SSRC), 5% lactic acid SRC (LASRC), 5% Na_2_CO_3_ SRC (SCASRC), NaCl SRC (SCSRC), CaCl_2_ SRC (CCSRC), FeCl_3_ SRC (FCSRC), sodium cholate SRC (SCHSRC), NaOH (pH 10) SRC (SHSRC), Na_2_CO_3_ (pH 10) SRC (SCABSRC) and SDS (pH 10) SRC (SDSSRC) values, and a Chopin SRC kit was used to measure the SRC value. SRCs of the oat flours increased when the solvents turned from neutral (water and NaCl) to acidic (5% lactic acid) or alkaline (5% Na_2_CO_3_, CaCl_2_, FeCl_3_, NaOH and pH 10 Na_2_CO_3_), and rose as the metal ion valencies of the metal salts (NaCl, CaCl_2_ and FeCl_3_) increased. The β-glucan contents were significantly positively correlated with the SCSRC (0.83**), CCSRC (0.82**), SCHSRC (0.80**) and FCSRC (0.78*). SRC measurements of β-glucan in oat flours revealed that the CCSRC values were related with β-glucan (0.64*) but not related with protein and starch. CaCl_2_ could therefore potentially be exploited as a reagent for β-glucan assay.

## 1. Introduction

β-Glucan, a predominant non-starch polysaccharide from the cell wall in cereal, is a d-glucose polymer linked by β-(1,3)(1,4)-glycosidic bonds, it belongs to a short-chain glucan with a low molecular weight ranging within 5.3–257.2 kDa [1,2,3,4]. As a type of water-soluble fiber, oat β-glucan is an unbranched linear polysaccharide and originates from the aleurone layer and subaleurone layers. The content of β-glucan in oat is around 4%, and the β-glucan in oat is comprised of glucopyranosyl units connected by 70% of β-(1,4) and 30% of β-(1,3)-glycosidic bonds [4]. β-(1,3)-linkage breaks up the uniform structure of oat β-d-glucan, allowing the formation of viscous solutions upon solubilization [5].

Currently, β-glucan is mainly determined by enzyme-catalyzing, flow injection analysis (FIA) using fluorescence spectroscopy and High Performance Liquid Chromatography (HPLC) [6,7,8]. For the enzyme catalyzing analysis, β-glucan is hydrolyzed into oligosaccharides by means of a β-glucan-specific hydrolase and then the oligosaccharides are hydrolyzed into glucoses by means of β-glucanase. The glucose is determined by means of oxidase and catalase, and the outcome is employed to calculate β-glucan content. Although β-glucan can be accurately determined by this method, it requires costly hydrolases specific to β-glucan and takes a long time [9]. In the FIA using fluorescence spectroscopy, a fluorescent substance can be specifically combined with β-glucan, thus increasing its own fluorescence intensity and the fluorescence intensity increment is quantitively related with β-glucan content within certain limits. However, this relation is affected by fluorescence-emitting intensities of the fluorescent substance as well as testing conditions. In the HPLC method, β-glucan is hydrolyzed into oligosaccharides by means of endo-(1,3)(1,4)-β-d-glucose hydrolase and the oligosaccharides are isolated using C18 column. The refractive index of the isolated oligosaccharides is measured with water as the mobile phase and then quantitively determined by HPLC. However, the HPLC method requires expensive analytical instruments and manual operation [10]. Therefore, it is especially important to develop a simple, fast and precise method for β-glucan assay. Solvent retention capacity of wheat flour is the amount of solvent which can be retained after centrifugation [11]. There are four solvents: deionized water, 50% sucrose, 5% Na_2_CO_3_ and 5% lactic acid, commonly adopted to measure flour solvent retention capacity (SRC), resulting in four evaluation indexes for wheat flour qualities. For wheat flours, glutelin is lactic acid SRC (LASRC)-related, damaged starch is CO_3_ SRC (SCASRC)-related, pentosan is sucrose SRC (SSRC)-related, and all flour compositions are water SRC (WSRC)-related [12].

Oat β-glucan possesses all physiological properties of common dietary fibers, such as water retention, chelating and absorption to cholesterol and bile acid molecules and improving environments for microbe communities in intestinal tract [13,14,15,16]. Based on the reports of Guo et al. [17] and Yamazaki et al. [18], β-glucan is capable of absorbing such metal ions as Na^+^ and Ca^2+^ and presents an increasing water retention capacity in a solution of which the pH is regulated from a neutral one to an acidic one.

In this study, we determined the retention capacities of oat flours in different solvents, elicited differences in the retention capacities among several solvents, investigated the relationships between solvent retention capacities and β-glucan contents, and screened proper solvents to predict β-glucan contents in oat flours. All these would contribute to the development of a quick method for β-glucan analysis.

## 2. Results and Discussion

### 2.1. Physical and Chemical Properties of Oat Flours

As shown in Table 1, β-glucan contents of the oat flours are averaged at 4.30%, with a varying range within 3.92%–4.79%. Among them, the flours of Bayou 9 (4.70%) and Baiyan 19 (4.79%) have higher β-glucan contents while Bayou 1 (3.92%), Baiyan 13 (4.05%) and Baiyan 15 (3.98%) have lower β-glucan contents. β-glucan content of Zhengmai 129 (0.28%) is significantly lower than those of oat flours. The average protein contents of oat flours is 14.89%, with a varying range within 12.47%–16.30%. Oat flours with higher protein contents are from Bayou 9 (16.30%), Baiyan 2 (16.05%) and Baiyan 15 (15.94%) while the lower protein content was from Baiyan 14 (13.99%). The wheat flour of Zhengmai 129 has the protein content of 12.47%, significantly lower than those of oat flours. The average starch content of oat flours is averaged at 57.74%, with a varying range within 54.61%–60.50%. Among the oat flours, Baiyan 14 (60.50%) and Bayou 9 (54.61%) have the highest and lowest starch contents, respectively. Zhengmai 129 has a starch content of 73.09%, significantly higher than those of oat flours.

Of the oat flours, the Calibrations of unit Chopin Dubois (UCDc) of damaged starch is averaged at 9.78, with a varying range within 4.35–12.80. Samples containing larger amounts of damaged starch (expressed by UCDc) are from Bayou 1 (12.80) and Bayou 12 (12.10) and that containing a lower amount is Baiyan 19 (4.35). Remarkably, UCDc of Zhengmai 129 (25.10) was significantly higher than those of oat flours (9.78). Particle size of oat flour (60–80 mesh sieve) is larger than that of wheat flour (100–120 mesh sieve), thus, damaged starch content of oat flour is lower than that of wheat flour. Damaged starch is defined as the starch of which surrounding cell membrane and granules are damaged by extruding, cutting, shearing, rubbing, ripping and other action forces in the process of milling [19]. In general, damaged starch granules can exert greater influence on flour qualities by absorbing several times more water than undamaged ones [20,21]. Native starch can absorb about 39%–87% water (by weight), while damaged starch is about 200%–430% [20]. Therefore, we measured the damaged starch for the purposes of better understanding the SRCs of oat flours with different components and analyzing the correlation between the components and their SRCs. Oat flour swelling analysis needs to take into account β-glucan, protein, starch, damaged starch contents as well as sample differences.

### 2.2. SRCs Against Different Solvents and Their Relations with Oat Flours Compositions

The WSRC, SSRC, LASRC, SCASRC values of oat flours were measured using conventional SRC measurements are summarized in Table 2. The LASRC, SCASRC, SSRC, WSRC values of oat flours are 132.78%, 110.71%, 91.46% and 90.12%, respectively. Flours with higher WSRC values are from Baiyan 2 (107.45%) and Bayou 9 (102.05%), while flours with lower WSRC values are from Bayou 1 (78.35%), Baiyan 14 (76.25%) and Zhengmai 129 (68.40%). Flours with higher SSRC values are from Zhengmai 129 (109.65%) and Baiyan 19 (96.75%), while flours with lower SSRC values are from Baiyan 14 (89.80%), Bayou 12 (89.20%), Bayou 9 (89.15%), Bayou 1 (88.40%), and Baiyan 13 (88.10%). Flours with higher LASRC values are from Baiyan 19 (159.30%) and Bayou 1 (154.15%), while the flour with the lower LASRC values is from Bayou 12 (85.95%). Flours with higher SCASRC values are from Bayou 9 (120.70%), Baiyan 2 (119.65%) and Baiyan 19 (118.75%), while the flour with the lower SCASRC value is from Zhengmai 129 (87.10%).

Modified SRC measurements were also used to identify the effects of metal ions, SRCs against different solvents are summarized in Table 2. The FeCl_3_ SRC (FCSRC), CaCl_2_ SRC (CCSRC), NaCl SRC (SCSRC), sodium cholate SRC (SCHSRC) values of oat flours are 157.09%, 99.65%, 88.21% and 64.13%, respectively. The flour with the higher SCSRC values are from Baiyan 9 (95.95%) and Baiyan 2 (95.60%), while the flour with the lowest SCSRC value is from Zhengmai 129 (70.70%). The flour with the highest CCSRC value is from Baiyan 2 (110.85%), and the flour with the lowest CCSRC value is from Zhengmai 129 (85.95%). The flour with the highest FCSRC value is from Bayou 9 (181.10%), and the flour with the lowest FCSRC value is from Zhengmai 129 (109.80%). The flour with the highest SCHSRC value is from Zhengmai 129 (77.45%), and the flour with the lowest SCHSRC value is from Bayou 1 (53.55%).

Modified SRC measurements were used to identify the effect of different solvent with the same pH, SRCs against different solvents are summarized in Table 2. The SHSRC, SCABSRC and SDSSRC values of oat flours are 91.74%, 91.70% and 72.01%, respectively. The flour with the highest SHSRC value is from Baiyan 2 (105.80%), and the flour with the lowest SHSRC value is from Zhengmai 129 (68.05%). The flour with the highest SCABSRC value is from Baiyan 2 (105.55%), and the flour with the lowest SCABSRC value is from Zhengmai 129 (67.70%). The flour with the highest SDSSRC value is from Baiyan 2 (80.05%), and the flour with the lowest SDSSRC value is from Zhengmai 129 (48.60%). With the three solutions kept at pH 10, the SHSRCs and SCABSRCs are consistent with each other and higher than SDSRC.

As shown in Table 2, the CV of the solvent retention capacities of the eight oat flours rank in the decreasing order of 5% lactic acid (19.18), water (12.71), sodium cholate (11.86), NaOH (pH 10) (11.47), Na_2_CO_3_ (pH 10) (11.38), CaCl_2_ (8.25), 5% Na_2_CO_3_ (8.13), FeCl_3_ (8.10), SDS (pH 10) (7.84), NaCl (6.77), 50% sucrose (3.89). The coefficient of variation (CV) is a statistic that measures the degree of variability in the data, and can determine whether there are significant differences among samples. Significant differences among samples can help expected the experimental errors.

With their solvent pH turned acid (lactic acid) or alkaline (Na_2_CO_3_), the SRCs of the oat flours slightly increased. Zhang [22] interpreted that hydrogen ions or hydroxyl ions could affect hydrogen bonds between dietary fiber molecules, resulting in partial breakage and combination of hydrogen ions inside fiber molecules with water and then increased WSRCs. With the lower ionic valencies of metal salt solvents, the SRCs of oat and wheat flours turn lower and are much higher than that against sodium cholate.

Because of the higher pentosan contained in wheat flour, the sucrose retention capacity is higher than those of oat flour [15], which follows the characterization of sample-contained pentosan in the standard SRC assay that SSRCs can indicate sample-contained pentosan. The SRCs of wheat flour against all the other solvents are lower than those of the oat flours, which can be attributed to the more dietary fibers dominated by β-glucan in oat flours [23].

In order to identify the effects of components in oat flours on SRCs, we conduct the coefficient of correlation between components and solvent retention capacities and summarize in Table 3. β-glucan contents are extremely significantly positively correlated with the SCSRC values (0.83**), CCSRC values (0.82**) and SCHSRC values (0.80**), and significantly positive to the FCSRC values (0.78*). Protein contents are extremely significantly positively correlated with SCABSRC values (0.85**), SHSRC values (0.83**), FCSRC values (0.82**) and WSRC values (0.77**) and significantly positive to that SDSSRC values (0.76*), SCASRC values (0.75*) and SCSRC values (0.72*). Starch contents are significantly negatively correlated with the FCSRC values (−0.69*). There is no significant correlation between damaged starch and SRCs.

Based on the report of Guo et al. [17], β-glucan in oat flour appeared to be correlated with three metal salts and could increase the SRC with lower solvent ionic valency for the ability of absorbing metal ions. The correlativity between β-glucan and cholate salts derives from the absorption of cholate salts.

### 2.3. SRCs and SRC Correlations with β-Glucan, Protein and Starch Additions

In order to find out SRC patterns of oat flour versus β-glucan, protein and starch, protein (with a content of 81.96%), β-glucan (with a content of 88.20%) and starch (with a content of 90.33%) were extracted from the oat flours and then added into the flours of Baiyan 2, Baiyan 15 and Bayou 9 (Table 4). The prepared Baiyan 15 flours are with the differentiated β-glucan contents of 3.98%, 4.95% and 5.91%, the differentiated protein contents of 15.94%, 17.89% and 19.95%, and the differentiated starch contents of 57.15%, 59.15% and 61.11%. The prepared Baiyan 2 flours are with the differentiated β-glucan contents of 4.33%, 5.29% and 6.30%, the differentiated protein contents of 16.05%, 18.01% and 20.02%, and the differentiated starch contents of 58.22%, 63.23% and 68.28%. The prepared Bayou 9 flours are with the differentiated β-glucan contents of 4.70%, 5.66% and 6.71%, the differentiated protein contents of 16.30%, 18.25% and 20.23%, and the differentiated starch contents of 54.61%, 59.60% and 64.65%.

With the addition of β-glucan, flours from the three oat varieties present the values of WSRC, SSRC, LASRC, SCASRC, CCSRC, SCHSRC that tended to increase slightly (Table 4). Similar increase is found in values of SCASRC, CCSRC and SCHSRC according to Table 2.

With more protein added, the flours tend to show varying but generally increasing SRCs (Table 4). The values of WSRC, SSRC, LASRC, CCSRC, SCHSRC (Table 2) are not stable, while the SCASRC values increase.

With more starch added, all the SRCs of the three oat flours tend to decrease (Table 4). However, oat flours with higher starch contents in Table 2 present high SRCs for the combined effects of β-glucan and protein [18,24]. The water holding capacity of starch is weaker than protein and β-glucan.

With both β-glucan and protein added at 2%, the former exerts significantly stronger influence on the SRCs than the latter (Table 4), indicating that β-glucan has the stronger ability of holding and absorbing water [18,25].

Based on the Pearson’s correlation coefficients in Table 5, the β-glucan content are significantly correlated with the CCSRC values (0.64*), while the protein contents are not correlated with the SRCs against all the six solvents and the starch contents are significantly negatively correlated with SCASRC values (−0.74*). It follows that with flour differences among the oat varieties excluded, β-glucan can be evaluated in terms of CCSRC values. Protein contents do not present proper SRC patterns. SRC deceases with the increase of starch content, eliminating the possibility of SRC against starch exerting influence on β-glucan.

In one word, the CCSRC values are only related with the β-glucan content and have no relation with the protein and starch contents. As a result, CaCl_2_ has the potential to be used as a β-glucan assay reagent for the solvent retention capacities method.

## 3. Materials and Methods

Eight oat varieties, including Baiyan 2, 13, 14, 15 and 19 were obtained by Baicheng Academy of Agricultural Sciences in Jilin Province, and Bayou 1, 9 and 12 were obtained by Zhangjiakou Academy of Agricultural Sciences in Hebei Province. One wheat variety, Zhengmai 129 were provided by Henan Provincial Academy of Agricultural Sciences. After the foreign matters removed, wheat and oat grains were milled into whole flours with lab mill (LM-85/40, SuiBang, Wuxi, China) and stored at 4 °C for future use. All other chemicals were reagent grade.

### 3.1. Sample and Solution Preparations

β-Glucan [26], protein, starch [27] were separately extracted from Baiyan 2 Oat flour, freeze dried, and stored in −4 °C freezer.

The following reagent solutions were prepared: 50% sucrose, 5% Na_2_CO_3_ and 5% lactic acid; NaOH, Na_2_CO_3_ and SDS with the pH of 10; and 1 M NaCl, CaCl_2_, FeCl_3_ and sodium cholate.

### 3.2. Determination of Compositions

β-Glucan was measured using a β-glucan assay kit (Megazyme, Wicklow, Ireland); moisture content was measured following approved method 44-15A [11]; total protein content was measured following approved method 46-12 [11]; total starch content was measured using a starch assay kit (Megazyme, Wicklow, Ireland); damaged starch content was measured using a damaged starch instrument (SDmatic, Chopin, Paris, France). Each sample was measured in triplicate.

### 3.3. SRC Measurement

#### 3.3.1. Conventional SRC measurements

WSRC, SSRC, LASRC and SCASRC values of eight oat flours and one wheat flour were done by conventional SRC measurements. SRC measurements were conducted using CHOPIN SRC machine (SRC, Chopin). The measurement procedure was conducted as follows: 5.000 ± 0.005 g flour samples were weighed and placed into 50 mL centrifuge tubes. Tubes and samples in combination were weighed using a balance and the 25 g required solutions were drawn into syringes. The tubes and syringes were placed in corresponding positions as indicated on touch screen. After the instrument was turned on, the solutions in the syringes were injected into the tubes and the shaking and static system was in operation. Afterwards, they were nonstop centrifuged at 1000 g for 15 min and allowed to gradually come to a standstill. Next, the tubes were drained upside down for 10 min, taken out and weighed. Finally, the SRCs were automatically calculated. The SRCs were calculated by the following formula [12]:

$$\mathrm{SRC}\left(\%\right)=\left(\frac{wet\;pellet\left(g\right)}{flour\left(g\right)}\times \frac{86}{100-flour\;moisture\left(\%\right)}\right)\times 100$$

In which wet pellet is the sample weight after centrifugation and draining. All SRC analyses were performed in triplicate and the coefficient of variation of the SRC value was less than 2.0%.

#### 3.3.2. Modified SRC Measurements

Modified SRC measurements keep the conventional SRC measurement procedure, but changed the solvents. SCSRC, CCSRC, FCSRC, SCHSRC, SHSRC, SCABSRC and SDSSRC values of eight oat flours and one wheat flour were measured in triplicate.

#### 3.3.3. SRC Measurements of Flours added β-Glucan, Protein and Starch

Flours of Baiyan 2, 15 and Bayou 9 were sampled, and the extracted β-glucan, protein and starch were added in them separately to increase the contents of β-glucan (β-glucan content was increased by 1% and 2%), protein (protein content was increased by 2% and 4%) and starch (starch content was increased by 5% and 10%) in flours; and WSRC, SSRC, LASRC, SCASRC, CCSRC and SCHSRC values were measured in triplicate.

### 3.4. Statistic Analysis

The values were provided as means ± standard deviation (SD). The statistical significance of the differences among the parameters was assessed using analysis of variance (ANOVA) by Statistical Analysis System software 9.2 (SAS Institute, Cary, NC, USA), and group means were considered to be significantly different at *p* < 0.01 and *p* < 0.05.

## 4. Conclusions

### 4.1. Effect of pH and Metal Ions on SRCs of Oat Flours

The SRCs of the oat flours increased when the solvents turned from neutral (Water and NaCl) to acidic (5% lactic acid) or alkaline (5% Na_2_CO_3_, CaCl_2_, FeCl_3,_ pH 10 NaOH and pH 10 Na_2_CO_3_), and rose as the metal ion valencies of the metal salts (NaCl, CaCl_2_ and FeCl_3_) decreased.

### 4.2. Correlations between SRCs and Oat Flours Compositions

The FCSRC, SCHSRC, CCSRC, SCSRC values were significantly positively or extremely significantly positively correlated with the β-glucan contents (0.78*, 0.80**, 0.82** and 0.83**); the SCSRC, SCASRC, SDSSRC, WSRC, FCSRC, SHSRC, SCABSRC values were significantly positively or extremely significantly positively correlated with the protein contents (0.72*, 0.75*, 0.76*, 0.77**, 0.82**, 0.83** and 0.85**); only the FCSRC values (−0.69*) were significantly negatively correlated with the starch contents; and the SRCs against all the solvents were not correlated with the damaged starch contents.

### 4.3. The Best Reagent for β-Glucan Assay

In the same individual oat samples with different β-glucan, protein and starch contents, the CCSRC values were significantly positively correlated with β-glucan (0.64*), but not related with protein (0.39) and starch (−0.39), so that it could be employed as being a reagent for β-glucan assay. This result provided a theoretical basis for fast β-glucan assay and laid down a better foundation for β-glucan used in food industry.

## Figures and Tables

**Table 1:** Determination of the compositions in oat and wheat flours.

Variety	β-Glucan (Dry Basis%)	Protein (Dry Basis %)	Starch (Dry Basis %)	Damaged Starch (UCDc)
Bayou1	3.92 ± 0.03 ^g^	14.75 ± 0.03 ^d^	58.29 ± 0.13 ^c^	12.80 ± 0.14 ^b^
Bayou9	4.70 ± 0.01 ^b^	16.30 ± 0.07 ^a^	54.61 ± 0.06 ^g^	10.05 ± 0.21 ^e^
Bayou12	4.37 ± 0.00 ^c^	15.39 ± 0.01 ^c^	57.60 ± 0.08 ^d^	12.10 ± 0.14 ^c^
Baiyan2	4.33 ± 0.01 ^c^	16.05 ± 0.04 ^b^	58.22 ± 0.08 ^c^	11.40 ± 0.00 ^d^
Baiyan13	4.05 ± 0.03 ^e^	14.68 ± 0.10 ^d^	57.37 ± 0.07 ^e^	8.15 ± 0.07 ^f^
Baiyan14	4.22 ± 0.00 ^d^	13.99 ± 0.01 ^f^	60.50 ± 0.08 ^b^	7.60 ± 0.14 ^g^
Baiyan15	3.98 ± 0.01 ^f^	15.94 ± 0.07 ^b^	57.15 ± 0.04 ^f^	11.75 ± 0.35 ^c,d^
Baiyan19	4.79 ± 0.03 ^a^	14.41 ± 0.10 ^e^	58.21 ± 0.11 ^c^	4.35 ± 0.07 ^h^
Zhengmai129 (Wheat)	0.28 ± 0.00 ^h^	12.47 ± 0.01 ^h^	73.09 ± 0.04 ^a^	25.10 ± 0.14 ^a^
Range (Oat flour)	3.92–4.79	12.47–16.30	54.61–60.50	4.35–12.80
Mean (Oat flour)	4.30	14.89	57.74	9.78
CV (Oat flour)	7.47	5.61	2.83	29.44

Data were listed as means ± standard deviations of triplicate measurements; Figures followed by different letters in the same column mean significant difference (Duncan’s method, *p* < 0.05); CV: coefficients of variance.

**Table 2:** Significant differences of solvent retention capacities (SRCs) against 11 solvents.

Variety	WSRC	SSRC	LASRC	SCASRC	SCSRC	CCSRC	FCSRC	SCHSRC	SHSRC	SCABSRC	SDSSRC
Bayou1	78.35 ± 0.21 ^g^	88.40 ± 0.99 ^e^	154.15 ± 0.78 ^b^	103.85 ± 0.21 ^e^	82.10 ± 0.14 ^f^	88.55 ± 0.64 ^f^	143.80 ± 0.28 ^g^	53.55 ± 1.06 ^f^	81.60 ± 0.28 ^g^	85.00 ± 0.57 ^e^	69.15 ± 0.07 ^c^
Bayou9	102.05 ± 1.06 ^b^	89.15 ± 0.07 ^de^	105.00 ± 0.57 ^g^	120.70 ± 0.14 ^a^	93.05 ± 0.21 ^b^	104.10 ± 0.28 ^c^	181.10 ± 0.99 ^a^	65.45 ± 0.35 ^c^	104.25 ± 0.07 ^b^	104.95 ± 0.50 ^a^	73.95 ± 1.34 ^b^
Bayou12	96.85 ± 0.07 ^c^	89.20 ± 0.14 ^d,e^	85.95 ± 0.50 ^h^	106.80 ± 0.71 ^d^	88.80 ± 0.71 ^c^	100.25 ± 0.50 ^d^	151.25 ± 0.21 ^d^	66.10 ± 0.28 ^c^	97.50 ± 0.42 ^c^	98.15 ± 1.06 ^b^	68.90 ± 0.71 ^c^
Baiyan2	107.45 ± 0.35 ^a^	95.30 ± 0.28 ^c^	134.15 ± 1.34 ^e^	119.65 ± 0.35 ^a,b^	95.60 ± 0.42 ^a^	110.85 ± 0.07 ^a^	166.10 ± 0.00 ^b^	73.30 ± 0.71 ^b^	105.80 ± 0.28 ^a^	105.55 ± 0.21 ^a^	80.05 ± 0.64 ^a^
Baiyan13	88.15 ± 0.50 ^e^	88.10 ± 0.42 ^e^	151.75 ± 0.92 ^c^	114.60 ± 0.14 ^c^	84.95 ± 0.21 ^d^	95.15 ± 1.20 ^e^	155.15 ± 0.35 ^c^	60.75 ± 0.35 ^d^	89.80 ± 0.57 ^e^	86.20 ± 0.28 ^d^	72.95 ± 0.92 ^b^
Baiyan14	76.25 ± 0.50 ^h^	89.80 ± 0.85 ^d^	131.80 ± 0.85 ^f^	96.00 ± 0.71 ^f^	81.90 ± 0.42 ^f^	100.25 ± 1.34 ^d^	145.50 ± 1.27 ^f^	59.50 ± 0.99 ^de^	76.80 ± 0.28 ^h^	76.70 ± 0.00 ^f^	63.00 ± 0.57 ^d^
Baiyan15	80.45 ± 0.64 ^f^	94.95 ± 0.35 ^c^	140.10 ± 1.27 ^d^	105.35 ± 0.64 ^d^	83.35 ± 0.21 ^e^	89.45 ± 0.50 ^f^	148.95 ± 1.20 ^e^	58.50 ± 0.71 ^e^	84.40 ± 0.28 ^f^	84.50 ± 0.28 ^e^	69.20 ± 0.71 ^c^
Baiyan19	91.40 ± 1.13 ^d^	96.75 ± 0.35 ^b^	159.30 ± 0.57 ^a^	118.75 ± 1.34 ^b^	95.95 ± 0.21 ^a^	108.60 ± 0.99 ^b^	164.85 ± 0.35 ^b^	75.90 ± 1.27 ^a^	93.80 ± 0.00 ^d^	92.55 ± 0.07 ^c^	78.85 ± 0.92 ^a^
Zhengmai129 (Wheat)	68.40 ± 0.00 ^i^	109.65 ± 0.78 ^a^	132.50 ± 0.00 ^ef^	87.10 ± 0.57 ^g^	70.70 ± 0.14 ^g^	85.95 ± 0.07 ^g^	109.80 ± 0.28 ^h^	77.45 ± 0.64 ^a^	68.05 ± 0.92 ^i^	67.70 ± 0.42 ^g^	48.60 ± 0.14 ^e^
Range (Oat flour)	76.25–107.45	88.40–96.75	85.95–159.30	96.00–120.70	81.90–95.95	88.55–110.85	143.80–181.10	53.55–75.90	76.80–105.80	76.70–105.55	63.00–80.05
Mean (Oat flour)	90.12	91.46	132.78	110.71	88.21	99.65	157.09	64.13	91.74	91.70	72.01
CV (Oat flour)	12.71	3.89	19.18	8.13	6.77	8.25	8.10	11.86	11.47	11.38	7.84

Data were listed as means ± standard deviations of triplicate measurements; Figures followed by different letters in the same column mean significant difference (Duncan’s method, *p* < 0.05); CV: coefficients of variance.

**Table 3:** Coefficient of correlation between components of oat flour and solvent retention capacity.

SRC	Content			
	β-Glucan	Protein	Starch	Damaged Starch
WSRC	0.60	0.77 **	−0.52	0.08
SSRC	0.33	−0.60	0.12	−0.30
LASRC	−0.29	−0.31	0.36	−0.40
SCASRC	0.62	0.75 *	−0.63	−0.23
SCSRC	0.83 **	0.72 *	−0.36	−0.30
CCSRC	0.82 **	0.46	−0.01	−0.46
FCSRC	0.78 *	0.82 **	−0.69 *	−0.24
SCHSRC	0.80 **	−0.30	−0.10	−0.47
SHSRC	0.62	0.83 **	−0.62	0.09
SCABSRC	0.60	0.85 **	−0.62	0.21
SDSSRC	0.52	0.76 *	−0.37	−0.24

Data were listed as means ± standard deviations of triplicate measurements; Figures with “*” mean significant difference (Duncan’s method, *p* < 0.05); Figures with “**” mean significant difference (Duncan’s method, *p* < 0.01).

**Table 4:** The influence of β-glucan content and protein content and starch content on SRC tests.

Composition	Variety	Content	WSRC	SSRC	LASRC	SCASRC	CCSRC	SCHSRC
β-Glucan	Baiyan15	3.98	80.45 ± 0.64 ^b^	94.95 ± 0.35 ^b^	140.10 ± 1.27 ^b^	105.35 ± 0.63 ^b^	89.45 ± 0.50 ^c^	58.50 ± 0.71 ^c^
		4.95	86.50 ± 0.57 ^a^	95.30 ± 0.14 ^b^	154.50 ± 0.57 ^a^	107.40 ± 0.28 ^a^	93.10 ± 0.42 ^b^	64.90 ± 0.28 ^b^
		5.91	87.50 ± 0.42 ^a^	100.00 ± 0.85 ^a^	155.90 ± 0.00 ^a^	107.90 ± 0.14 ^a^	99.50 ± 0.00 ^a^	67.30 ± 0.14 ^a^
	Baiyan2	4.33	107.45 ± 0.35 ^c^	95.30 ± 0.28 ^c^	134.15 ± 1.34 ^a^	119.65 ± 0.35 ^b^	110.85 ± 0.07 ^c^	73.30 ± 0.71 ^b^
		5.29	108.30 ± 0.14 ^b^	96.70 ± 0.57 ^b^	136.20 ± 0.14 ^a^	120.70 ± 0.57 ^b^	113.80 ± 0.14 ^b^	74.40 ± 0.42 ^b^
		6.30	112.15 ± 0.07 ^a^	105.10 ± 0.28 ^a^	136.25 ± 0.07 ^a^	124.90 ± 0.00 ^a^	115.30 ± 0.28 ^a^	76.70 ± 0.14 ^a^
	Bayou9	4.70	102.05 ± 1.06 ^b^	89.15 ± 0.07 ^c^	105.00 ± 0.57 ^b^	120.70 ± 0.14 ^c^	104.10 ± 0.28 ^c^	65.45 ± 0.35 ^b^
		5.66	114.00 ± 0.99 ^a^	90.80 ± 0.14 ^b^	109.10 ± 0.14 ^a^	121.50 ± 0.28 ^b^	113.70 ± 0.71 ^b^	66.40 ± 0.57 ^b^
		6.71	115.10 ± 0.42 ^a^	99.30 ± 0.28 ^a^	109.90 ± 0.14 ^a^	127.60 ± 0.14 ^a^	119.80 ± 0.42 ^a^	69.70 ± 0.28 ^a^
Protein	Baiyan15	15.94	80.45 ± 0.64 ^b^	94.95 ± 0.35 ^a^	140.10 ± 1.27 ^a^	105.35 ± 0.64 ^b^	89.45 ± 0.50 ^b^	58.50 ± 0.71 ^b^
		17.89	81.90 ± 0.14 ^a^	89.00 ± 0.14 ^b^	114.80 ± 0.28 ^b^	106.50 ± 0.28 ^b^	91.00 ± 0.28 ^b^	60.90 ± 0.14 ^a^
		19.95	82.50 ± 0.28 ^a^	89.20 ± 0.28 ^b^	110.10 ± 0.85 ^c^	111.90 ± 0.28 ^a^	100.70 ± 0.71 ^a^	61.30 ± 0.57 ^a^
	Baiyan2	16.05	107.45 ± 0.35 ^b^	95.30 ± 0.28 ^a,b^	134.15 ± 1.34 ^a,b^	119.65 ± 0.35 ^c^	110.85 ± 0.07 ^c^	73.30 ± 0.71 ^b^
		18.01	110.60 ± 0.14 ^a^	94.80 ± 0.42 ^b^	135.40 ± 0.14 ^a^	122.10 ± 0.57 ^b^	112.80 ± 0.10 ^a^	76.90 ± 0.57 ^a^
		20.02	111.50 ± 0.57 ^a^	95.90 ± 0.14 ^a^	132.10 ± 0.28 ^b^	129.50 ± 0.42 ^a^	111.80 ± 0.28 ^b^	77.10 ± 0.14 ^a^
	Bayou9	16.30	102.05 ± 1.06 ^b^	89.15 ± 0.07 ^a^	105.00 ± 0.57 ^a^	120.70 ± 0.14 ^b^	104.10 ± 0.28 ^b^	65.45 ± 0.35 ^b^
		18.25	106.90 ± 0.14 ^a^	73.90 ± 0.57 ^b^	87.30 ± 0.28 ^b^	120.50 ± 0.14 ^b^	116.90 ± 0.57 ^a^	70.70 ± 0.28 ^a^
		20.23	108.80 ± 0.28 ^a^	70.80 ± 0.71 ^c^	85.50 ± 0.14 ^c^	122.10 ± 0.14 ^a^	115.70 ± 0.28 ^a^	71.20 ± 0.14 ^a^
Starch	Baiyan15	57.15	80.45 ± 0.64 ^a^	94.95 ± 0.35 ^a^	140.10 ± 1.27 ^b^	105.35 ± 0.64 ^a^	89.45 ± 0.50 ^a^	58.50 ± 0.71 ^a^
		59.15	70.00 ± 0.14 ^b^	84.60 ± 0.28 ^b^	149.10 ± 0.14 ^a^	95.80 ± 0.57 ^b^	89.10 ± 0.14 ^a^	54.70 ± 0.14 ^b^
		61.11	68.80 ± 0.42 ^b^	83.00 ± 0.14 ^c^	123.80 ± 0.28 ^c^	93.30 ± 0.14 ^c^	80.20 ± 0.00 ^b^	54.70 ± 0.00 ^b^
	Baiyan2	58.22	107.45 ± 0.35 ^a^	95.30 ± 0.28 ^a^	134.15 ± 1.34 ^a^	119.65 ± 0.35 ^a^	110.85 ± 0.07 ^a^	73.30 ± 0.71 ^a^
		63.23	100.00 ± 0.28 ^b^	92.50 ± 0.57 ^b^	133.00 ± 0.28 ^a^	105.80 ± 0.14 ^b^	108.70 ± 0.57 ^b^	69.80 ± 0.57 ^b^
		68.28	70.60 ± 0.14 ^c^	84.00 ± 0.14 ^c^	110.30 ± 0.14 ^b^	102.20 ± 0.28 ^c^	100.30 ± 0.14 ^c^	59.00 ± 0.14 ^c^
	Bayou9	54.61	102.05 ± 1.06 ^a^	89.15 ± 0.07 ^a^	105.00 ± 0.57 ^a^	120.70 ± 0.14 ^a^	104.10 ± 0.28 ^a^	65.45 ± 0.35 ^a^
		59.60	90.60 ± 0.85 ^b^	83.10 ± 0.14 ^b^	91.40 ± 0.14 ^b^	103.30 ± 0.28 ^b^	102.60 ± 0.42 ^b^	55.80 ± 0.57 ^b^
		64.65	89.90 ± 0.00 ^b^	81.90 ± 0.42 ^c^	89.30 ± 0.42 ^c^	100.10 ± 1.41 ^c^	90.70 ± 0.42 ^c^	54.70 ± 0.28 ^b^

Data were listed as means ± standard deviations of triplicate measurements; Figures followed by different letters in the same column mean significant difference (Duncan’s method, *p* < 0.05).

**Table 5:** Pearson’s correlation coefficients between flour constituent levels and SRC values of eight oat flours.

SRC	Content		
	β-Glucan	Protein	Starch
WSRC	0.54	0.20	−0.62
SSRC	0.59	−0.42	−0.60
LASRC	−0.16	−0.42	−0.09
SCASRC	0.53	0.37	−0.74 *
CCSRC	0.64 *	0.39	−0.39
SCHSRC	0.49	0.28	−0.41

Figures with “*” mean significant difference (Duncan’s method, *p* < 0.05).

## Abbreviations

SRC	Solvent retention capacity
WSRC	Water SRC
SSRC	50% Sucrose SRC
LASRC	5% Lactic acid SRC
SCASRC	5% Na_2_CO_3_ SRC
SCSRC	NaCl SRC
CCSRC	CaCl_2_ SRC
FCSRC	FeCl_3_ SRC
SCHSRC	Sodium cholate SRC
SHSRC	NaOH(pH 10) SRC
SCABSRC	Na_2_CO_3_(pH 10) SRC
SDSSRC	SDS(pH 10) SRC
HPLC	High Performance Liquid Chromatography
FIA	Flow injection analysis
UCDc	Calibrations of unit Chopin Dubois
